# Structural Communication Between C-Peptide and Insulin Within the Proinsulin Molecule

**DOI:** 10.3390/ijms27010483

**Published:** 2026-01-02

**Authors:** Rubing Shao, Maroof Alam, Leena Haataja, Peter Arvan

**Affiliations:** 1 Division of Metabolism, Endocrinology & Diabetes, University of Michigan, Brehm Tower rm 5112, 1000 Wall Street, Ann Arbor, MI 48105, USA; rubings@umich.edu (R.S.); mdal@umich.edu (M.A.);; 2 Department of Molecular & Integrative Physiology, University of Michigan, Ann Arbor, MI 48105, USA

**Keywords:** protein folding, disulfide bonds, endoplasmic reticulum, protein trafficking, protein processing, insulin secretion

## Abstract

Despite years of study, the biological role of the human proinsulin connecting peptide (C-peptide) remains poorly understood. Nevertheless, the C-peptide exhibits subdomains including conserved residues that are thought to have co-evolved with the insulin moiety of proinsulin. Genome-wide association studies in humans suggest that alterations of glycemic control may exhibit a possible linkage with the presence of certain C-peptide variants other than frame-shifts, stop codons, alternative splice variants, or the addition of an extra unpaired Cys residue. Although the C-peptide is ultimately excised from insulin, here, we have bioengineered missense mutations in the amino-terminal portion of the C-peptide (especially involving or near preproinsulin residues Q62,V63) that we find impair proinsulin folding and trafficking efficiency and, in this way, impair insulin biogenesis. We show that proinsulin bearing a C-peptide missense variant can also physically interact with co-expressed wildtype proinsulin, affecting the trafficking behavior of both proinsulin proteins in a manner that is directly related to the relative expression ratio of the variant and wildtype gene products. We conclude that in addition to other possible functions, the amino-terminal portion of the C-peptide influences proinsulin folding and trafficking and, in this way, affects human insulin production.

## 1. Introduction

Recently, hundreds of chromosomal loci bearing single-nucleotide polymorphisms associated with type 2 diabetes (T2D) have been grouped into gene clusters [1,2]. These categories are generally consistent with those originally described by Groop and colleagues, which led to the proposal that “pancreatic islet β-cell function (adjusted for insulin resistance) is the strongest predictor of future diabetes” [3]. Of the many islet-related gene variants linked to random (i.e., non-fasting) blood glucose and diabetes, a number of them influence the biosynthesis and trafficking of proinsulin, including the *INS* gene itself [4]. Interestingly, whereas heterozygous mutations that impair transcription from the affected *INS* allele can contribute to diabetes risk, the inheritance of such mutations is recessive because insulin production from the expression of a single wildtype *INS* allele is sufficient to preserve blood glucose homeostasis [5]. However, many heterozygous *INS* gene mutations that alter the proinsulin coding sequence can cause diabetes by triggering proinsulin misfolding, which may exhibit dominant-negative gain-of-toxic-function [6]—a syndrome sometimes referred to as Mutant *INS*-gene-induced Diabetes of Youth (MIDY [7], also known as MODY10 [8]).

Wildtype proinsulin itself is said to have evolved “at the edge of foldability” [9], and in hyperphagic animals such as *db*/*db* mice, insulin resistance linked to nutritional excess (accompanied by compensatory upregulation of insulin synthesis) drives increased proinsulin misfolding in the endoplasmic reticulum (ER), despite expressing only wildtype *INS* gene products [10,11,12,13]. Further, proinsulin misfolding inhibits the efficiency of its anterograde trafficking, thereby contributing to both β-cell ER stress [14] and relative insulin deficiency [15,16]—features that are described in human T2D [14,17,18] and that can be improved upon treatment [19]. There are many *INS* gene variants generating a range of clinical phenotypes from permanent neonatal diabetes to clinically silent [20]. Recent findings have suggested that MIDY variants with even a subtle predisposition to proinsulin misfolding provide a risk factor for the development of diabetes provoked by nutritional excess [21]. It is likely that rare patients carrying a clinical diagnosis of T2D unknowingly bear heterozygous mild MIDY variants that contribute to disease pathogenesis despite receiving clinical treatment indistinguishable from others with T2D [22], including approaches in which exogenous insulin is not used [23,24].

The C-peptide [25] has evolved to assist proinsulin [26,27] in the formation of mature insulin [27,28] and has been described as one of the most under-appreciated components of insulin biology [29]. The C-peptide “presents” proinsulin dibasic sites for prohormone convertase-mediated cleavage at the B-chain/C-peptide and C-peptide/A-chain junctions [30,31] followed the action of carboxypeptidase E [32]. The purified 31-residue C-peptide (derived from human preproinsulin residues 57–87) is said to be intrinsically disordered in buffered solution [33,34]; indeed, it is possible to dispense with certain C-peptide residues (and even endoproteolytic cleavage) while retaining some ability to generate a secreted protein that is capable of activating insulin receptors [35]. However, within proinsulin, C-peptide contains several subdomains with conserved structural features [36,37].

Whereas certain mutations in insulin B- or A-chain sequences [20] are known to induce MIDY [38], mutations landing within the C-peptide are less commonly diabetogenic and are limited mostly to mutations in flanking cleavage sites (i.e., immediately outside of the C-peptide), stop codons, frame-shifts, splice variants that eliminate the insulin A-chain (which is absolutely required for disulfide pairing with the B-chain to allow for proinsulin export from the ER), or missense mutants generating a novel Cys residue (G69C, G75C, or S85C [20,39]), all of which likely interfere with native proinsulin disulfide maturation. Nevertheless, within the human T2D-GWAS portal (https://t2d.hugeamp.org), a number of *INS* missense variants landing within the C-peptide can be found [4], some of which might alter proinsulin folding to the native state. Thus far, three non-Cys missense substitutions have been reported in publication: G84R and L68M were deemed of uncertain pathogenicity [40], and a 52 year-old man carrying a diagnosis of T2D has been described with heterozygous expression of proinsulin-Q65R [22]. However, direct studies were not performed on the latter variant, nor was there genetic testing of diabetic first-degree relatives [22].

**Figure 1:**
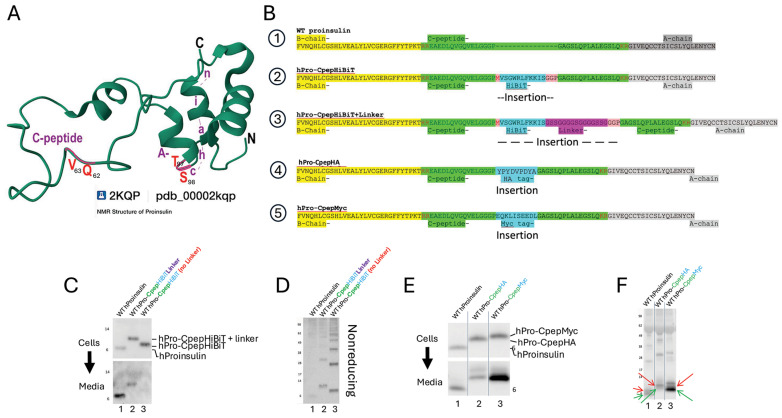
Structure of the native and epitope-tagged C-peptide. (**A**) NMR structure of a bioengineered monomer human proinsulin (https://www.rcsb.org/3d-view/2KQP). (**B**) Five distinct sequences of human WT proinsulin epitope-tagged within the C-peptide. B-chain, yellow; C-peptide, green; extra sequences, peach, blue, and fuchsia; A-chain, gray. (**C**) 293T cells expressing WT human proinsulin or that bearing HiBiT ± linker. Media bathing-transfected cells were collected overnight; Western blotting shows intracellular and secreted proinsulin for each construct. (**D**) Cell lysates from (**C**) were resolved by non-reducing SDS-PAGE. In this panel, the gel was not treated with DTT and heating before electrotransfer and Western blotting with anti-human proinsulin. (**E**) Reducing SDS-PAGE and immunoblotting with anti-proinsulin of media and 293T cells transfected to express the indicated proinsulin constructs. (**F**) Cell lysates and media from (**E**) were combined (for total proinsulin) and resolved by non-reducing SDS-PAGE. After electrotransfer, the membrane was probed with anti-human proinsulin. Monomeric proinsulin included faster migrating forms that have native disulfide bonds (green arrows) and slower migrating bands with non-native disulfide bonds (red arrows).

If proinsulin were to undergo successful endoproteolytic cleavage, the C-peptide missense variants would ultimately not be contained within the final structure of mature insulin itself. However, preproinsulin-Q62 within the C-peptide, largely conserved in mammals, has been described as a crucial residue suggested to interact with the insulin A-chain [41], and in the process of evolutionary selection of proinsulin structure, this residue is positively correlated with presence of A-chain residues T97 and S98 (Figure 1A)—together described as co-evolution [42]. Here, we have generated several missense substitutions in this C-peptide subdomain and generated the recently described Q65R variant [22] in order to examine the contribution of this portion of C-peptide to the folding and trafficking of the parent proinsulin molecule.

## 2. Results

### 2.1. The Folding of Proinsulin Exhibits Sequence-Specific Sensitivity to Changes in C-Peptide Structure, Which Controls the Efficiency of Proinsulin Anterograde Trafficking

Studies of recombinant proinsulin have frequently used epitope-tagging of the C-peptide, including the introduction of Flag, V5, myc, and His epitope-tags [10], as well as GFP [43] and other fluorescent proteins [44], or enzymatic activities such as luciferase [45] or others [12]. Because the glycine-rich central portion of the C-peptide offers peptide flexibility and is both spatially separated from the insulin chains (Figure 1A) and ultimately cleaved from mature insulin, this region has generally been considered as a site impervious to the introduction of foreign polypeptide sequences. Here, we compared several distinct epitope-tagged proinsulins expressed recombinantly in 293T cells. HiBiT [46] was introduced either as a 15- or 30-amino acid insertion (i.e., ±a linker peptide [47]); HA as a 9-residue insertion; and myc as a 10-residue insertion, each at the same entry point within the C-peptide (Figure 1B). 293T cells cannot convert proinsulin to insulin; thus, cells (and overnight culture media) could be analyzed to directly examine the efficiency of the forward-trafficking of proinsulin from transfected cells to media. In comparison to untagged WT proinsulin (Figure 1C lane 1), HiBiT-tagged human proinsulin, with or without the linker peptide, was secreted inefficiently (Figure 1C lanes 2 + 3). Analysis of cell lysates by proinsulin immunoblotting after non-reducing SDS-PAGE showed a predisposition of HiBiT-tagged proinsulins to accumulate intracellularly as aberrant intermolecular disulfide-linked complexes (Figure 1D). Whereas proinsulin trafficking was also impaired upon introduction of an HA-tag, myc-tagged proinsulin exhibited no such defect (Figure 1E). After non-reducing SDS-PAGE, we treated the gel with reducing agent (and heating) before electrotransfer and immunoblotting [11] to improve the detection of proinsulin bearing native disulfide bonds (Figure 1F green arrows) and non-native proinsulin monomers (red arrows). A decreased fraction of native monomers (Supplemental Figure S1) correlates with diminished secretion efficiency (e.g., hPro-CpepHA, Figure 1E). These data indicate that the folding and trafficking of proinsulin exhibits sequence-specific sensitivity to changes in C-peptide structure.

**Figure 2:**
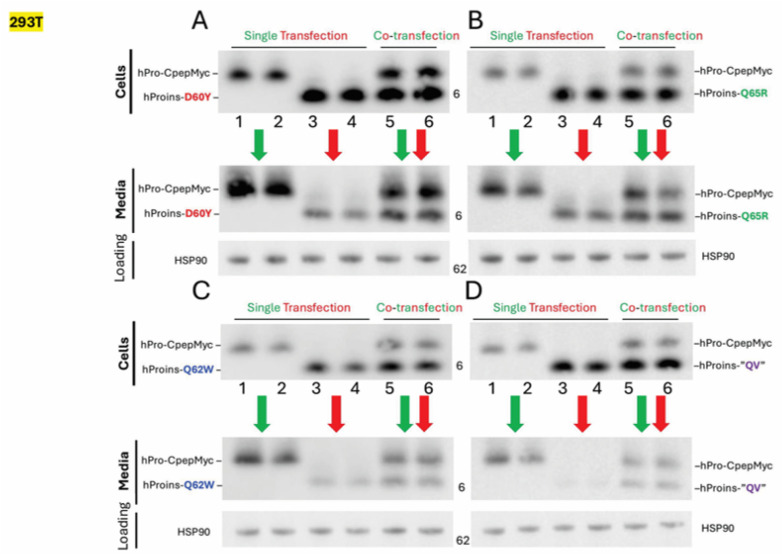
C-peptide variant proinsulins exhibit reduced secretion efficiency under single-transfection conditions, while co-expression with WT proinsulin enhances secretion of the variants. 293T cells were transfected with a fixed total amount of plasmid (single transfections included empty vector to maintain the same total plasmid DNA). WT human proinsulin was myc-epitope-tagged (upper band); proinsulin variants were untagged (lower bands). Efficient secretion of single-transfected hPro-CpepMyc is shown with a single downward green arrow. Inefficient variant secretion is shown with a single downward red arrow. Secretion upon co-expression with hPro-CpepMyc is shown with a downward double (red/green) arrow. HSP90 is a loading control. Panels (**A**–**D**) each show a different proinsulin variant, as indicated. Note that the “QV” in panel D is the double substitution Q62W,V63R.

**Figure 3:**
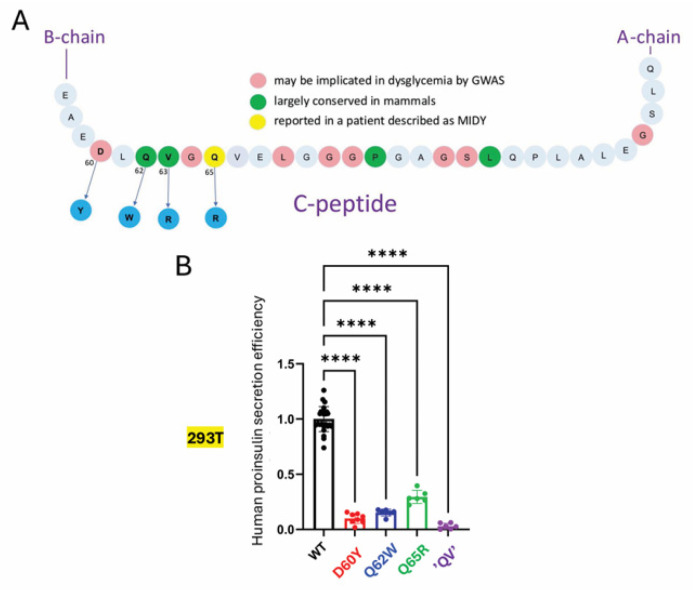
Defective secretion of C-peptide variant proinsulins in 293T cells. (**A**) Primary structure of the human proinsulin C-peptide, highlighting residues of interest, and four residues mutated in this study (in blue). (**B**) Quantitation of secretion efficiency (proinsulin in media/proinsulin in cells) from replicate experiments like those shown in lanes 1–4 of Figure 2. The results for all human proinsulin C-peptide variants are normalized to those of WT hPro-CpepMyc (*n* = 6 independent experiments; mean ± SD; **** *p* < 0.0001).

The T2D GWAS portal includes *INS* gene SNPs of uncertain significance that encode several missense substitutions affecting residues in the region from D60–Q65 that are contained within the amino-terminal portion of the C-peptide emerging from a β-turn [36]. Several of these residues are said to have co-evolved with residues of the insulin A-chain [41,42], although a stable physical contact is not apparent from the spatial relationship found in the NMR structure of a bioengineered monomeric proinsulin (Figure 1A, [27]). To further explore this subdomain, we introduced four missense variants into human proinsulin (Figure 2 and Figure 3A). Upon expression in 293T cells, diminished secretion efficiency in comparison to hPro-CpepMyc was observed for substitutions D60Y (Figure 2A lanes 1–4), Q65R (Figure 2B lanes 1–4), Q62W (Figure 2C lanes 1–4), or a double-mutant known as “QV” (Q62W,V63R; Figure 2D lanes 1–4). From replicate experiments, these results were quantified in Figure 3B. These data further support that the folding and trafficking efficiency of proinsulin is sensitive to specific sequence changes within the C-peptide structure.

### 2.2. MIDY-like Behavior of Proinsulin C-Peptide Missense Variants

**Figure 4:**
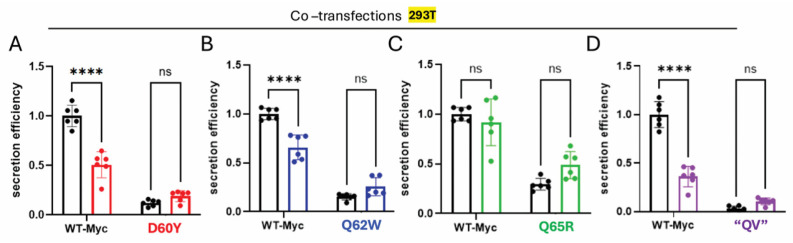
Co-expression of WT hProCpep-Myc with C-peptide variant proinsulins lowers the secretion efficiency of the WT partner. (**A**–**D**) Quantitation of secretion efficiency (proinsulin in media/proinsulin in cells) from replicate experiments like those shown in lanes 5–6 of Figure 2. The black bars reflect secretion efficiency under single-transfection conditions; the colored bars show the secretion efficiency of each construct under co-transfection conditions. The results for all constructs are normalized to those of WT hPro-CpepMyc under single-transfection conditions (*n* = 6 independent experiments; mean ± SD; **** *p* < 0.0001; ns = not significant).

**Figure 5:**
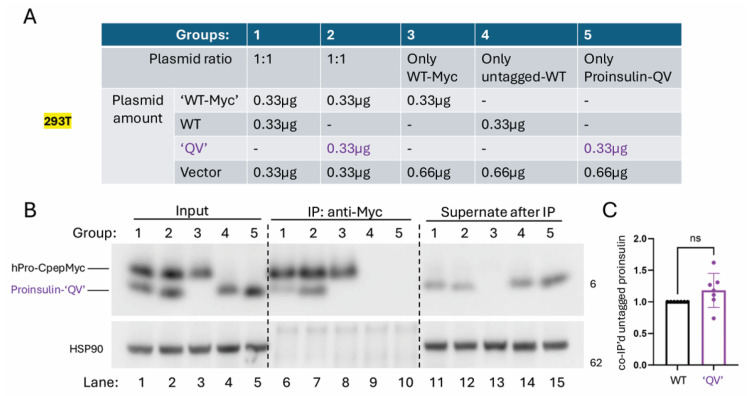
Despite being defective for secretion, the human proinsulin C-peptide variant “QV” exhibits a normal level of physical interaction with co-expressed WT proinsulin. (**A**) 293T cells were transfected with the indicated plasmids. Vector is empty vector so that all samples are transfected with same amount of total plasmid DNA. (**B**) Immunoprecipitation with anti-myc efficiently recovers hPro-CpepMyc (almost none of which remains behind in the supernate after IP). Additionally, IP of hPro-CpepMyc co-precipitates untagged WT or C-peptide proinsulin variants. (**C**) Quantitation of the fraction of untagged input proinsulin that is co-precipitated with hPro-CpepMyc, normalized to the result obtained with untagged WT human proinsulin (*n* = 7 independent experiments; mean ± SD; ns = no significant difference in co-IP efficiency).

Because hPro-CpepMyc undergoes a folding and trafficking process that is at least as efficient as that of WT human proinsulin yet has a slower electrophoretic gel mobility, we were able to co-transfect hPro-CpepMyc with the missense variants and follow both proteins simultaneously. In each case, secretion of the missense variant improved in cells co-transfected with the WT proinsulin construct (Figure 2A–D, media; compare lanes 5 + 6 to 3 + 4). From replicate experiments, these results are quantified in Figure 4. The data suggest that properly folded proinsulin may offer partial rescue to co-expressed proinsulin C-peptide variants, just as it does for some mild MIDY mutants [48]. Properly folded proinsulin is known to non-covalently homodimerize in the ER prior to trafficking to the Golgi complex [49]. We therefore sought to compare the extent to which properly folded hPro-CpepMyc could physically interact with untagged WT proinsulin control or “QV” as a representative C-peptide variant. For this, we immunoprecipitated from transfected cells (Figure 5A) with anti-myc antibodies that can only recognize hPro-CpepMyc (Figure 5B; compare lanes 9 + 10 to 6–8). Despite its secretory defect, the “QV” C-peptide variant was not defective for co-immunoprecipitation with co-expressed WT hPro-CpepMyc (Figure 5B lane 7, quantified in Figure 5C). The data suggest that secretion rescue involves physical interaction (likely to be non-covalent dimerization) between co-expressed WT proinsulin and C-peptide variants, which might limit the genetic penetrance of such variants in genome-wide association studies in humans with diabetes and β-cell dysfunction (and related glycemic control phenotypes).

**Figure 6:**
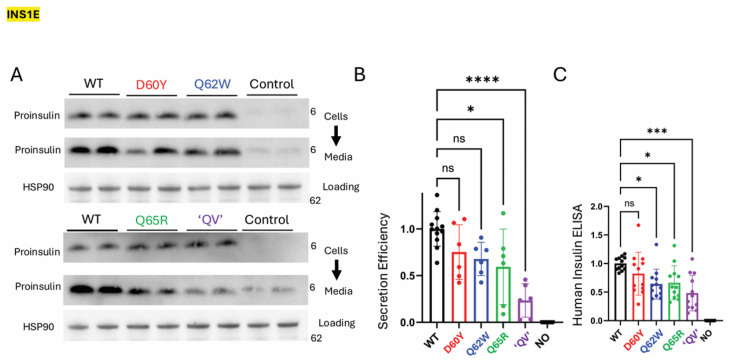
Secretion of human C-peptide variants and formation of human insulin in INS1E (rat pancreatic) β-cells. INS1E cells were transfected to express untagged WT human proinsulin or each of the four human C-peptide variants. Because of the weak cross-reactivity of rat proinsulin with human-specific proinsulin antibodies, a set of untransfected cells (“Control”) was processed in parallel. (**A**) Western blotting of cell lysates and an overnight collection of media (the same fraction of cells and media as shown). HSP90 is a loading control. (**B**) Quantitation of secretion efficiency (proinsulin in media/proinsulin in cells) of human proinsulin C-peptide variants expressed in INS1E cells, normalized to that obtained for untagged WT human proinsulin (*n* = 6 independent experiments; mean ± SD; * *p* < 0.05; **** *p* < 0.0001; ns = not significant). (**C**) ELISA detecting mature human insulin production in INS1E cells transfected as in panels A + B (*n* = 12 independent experiments; mean ± SD; * *p* < 0.05; *** *p* < 0.001; ns = not signfiicant).

To explore this question in β-cells that express abundant endogenous proinsulin, we expressed the same C-peptide variants in the INS1E rat pancreatic β-cell line (which does process some proinsulin to insulin but also robustly secretes unprocessed proinsulin to the culture medium—reflecting successful proinsulin export through the secretory pathway [38]). The total proinsulin recovery of human C-peptide missense variants was less than that of WT proinsulin in both 293T cells and INS1E cells (and we normalized to human INS mRNA to ensure that levels were not attributed to differences in transient transfection efficiency between samples; see Supplemental Figure S2). Nevertheless, in INS1E cells, expression of human C-peptide missense variants in these cells (Figure 6A) did not even show a statistically significant decrease in secretion efficiency for human proinsulin D60Y and Q62W variants (detected by immunoblotting with human-specific anti-proinsulin antibody). The Q65R variant achieved the lowest level of statistical significance; the QV variant was the only one that still showed markedly inhibited secretion efficiency (Figure 6B), and its intracellular distribution did not concentrate in the juxtanuclear Golgi region like that of WT proinsulin (Supplemental Figure S3). Conceivably, decreased secretion efficiency of C-peptide variant proinsulins could have occurred if such variants were to exhibit enhanced conversion from proinsulin-to-insulin; however, this was clearly not the case; none of the variants yielded increased human insulin (measured by human-specific insulin ELISA) compared to that generated from WT human proinsulin (Figure 6C). Indeed, the decrease in human insulin generation from each C-peptide variant corresponded rather closely with the diminished efficiency of human proinsulin secretion (compare Figure 6B,C). Thus, it appears that neither the β-cell-specific ER folding environment nor the ongoing synthesis of a large excess of endogenous WT proinsulin is enough to fully rescue the defective trafficking of the human proinsulin C-peptide “QV” variant.

### 2.3. The Relative Protein Expression Ratio of WT to Variant Proinsulin Influences the Trafficking Phenotype

The above co-expression studies involving C-peptide variants also suggested the possibility of diminished secretion efficiency of co-expressed non-mutant proinsulin (hPro-CpepMyc, Figure 2A–D Media lanes 5 + 6 compared to lanes 1 + 2). Replicates of this experiment are quantified in Figure 4. This behavior is reminiscent of that exhibited by authentic MIDY mutants that impair the trafficking of co-expressed WT proinsulin, a phenotype that is central to the onset of diabetes in MIDY [6,7]. Interestingly, of all the C-peptide variants tested, the Q65R C-peptide variant (suggested as a diabetogenic MIDY variant [22]) produced no significant dominant-negative effect on the trafficking of co-expressed WT hPro-CpepMyc (Figure 4C), raising questions about whether this monogenic allele can truly function as a dominant-negative. However, each of the other C-peptide variants tested did appear inhibitory to WT proinsulin trafficking, with the “QV” variant yielding the greatest effect (Figure 4A,B,D).

**Figure 7:**
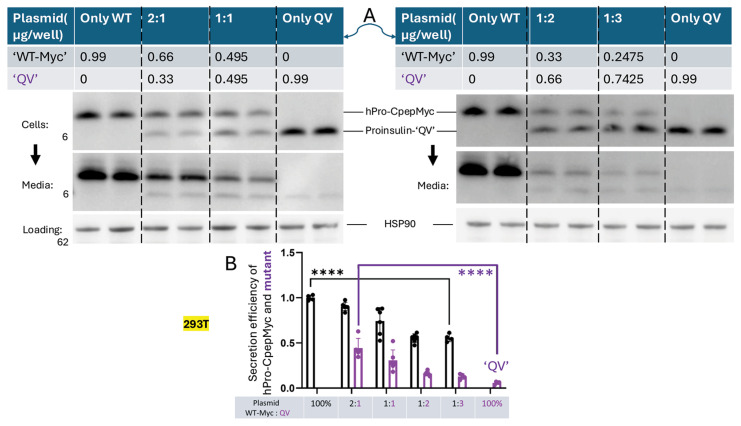
Varying the ratio of co-expressed WT and variant proinsulin partners changes the secretion efficiency of both partners. (**A**) 293T cells were co-transfected with plasmid in the ratios indicated at the top of the panel. Cells and media collected overnight were immunoblotted with anti-human proinsulin. The upper band in each lane is WT hPro-CepMyc, and the lower band in the human proinsulin “QV” variant, each expressed at different levels. (**B**) Quantitation of secretion efficiency of each partner from replicate experiments like that shown in panel (**A**) (*n* = 4 independent experiments; mean ± SD; **** *p* < 0.0001).

Mice express four functional *Ins* gene alleles, humans only two. We tested the relative abundance (ratio) of WT-to-variant proinsulin [48] and observed that an increasing fraction (and even an increased absolute amount) of the “QV” variant was secreted even when less plasmid DNA encoding the variant was expressed but co-transfected with a larger amount of WT proinsulin partner (Figure 7A, quantified in Figure 7B). This behavior was not observed when both untagged and tagged proinsulin partners comprised WT proinsulin (Supplemental Figure S4). The data suggest that rescue of the variant proinsulin product is greater when an increased fraction of WT proinsulin is co-expressed, as might be the case in rodents, bearing three WT alleles, compared to humans, bearing only one other WT allele. 

**Figure 8:**
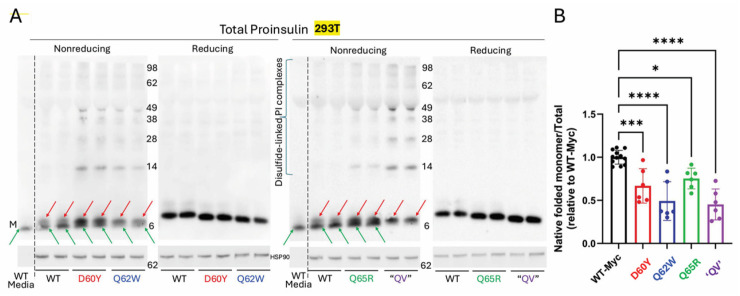
Human C-peptide variants exhibit increased proinsulin misfolding. (**A**) Each cell lysate was combined with culture media collected overnight to recover total proinsulin. After non-reducing SDS-PAGE and transferring to nitrocellulose, immunoblotting of human C-peptide variants showed an increased fraction of proinsulin monomers bearing non-native disulfide bonds (red arrows) plus aberrant intermolecular disulfide-linked proinsulin complexes (higher-molecular-weight bands). Proinsulin bearing native disulfide bonds is highlighted with green arrows. (**B**) Quantitation of the percentage of native disulfide-bonded proinsulin as a fraction of all proinsulin recovered upon reducing SDS-PAGE (*n* = 6 independent experiments; mean ± SD; * *p* < 0.05; *** *p* < 0.001; **** *p* < 0.0001).

To explore the basis for the secretion deficiency of human proinsulin C-peptide variants, we examined the folding status of each construct by non-reducing SDS-PAGE with post-gel disulfide reduction, followed by immunoblotting with anti-human-proinsulin [11]. Each C-peptide variant (shown in independent duplicates as well as reducing gels, Figure 8A) exhibited an increase in aberrant intermolecular disulfide-linked complexes of proinsulin (ranging from dimers that ran near the 14 kDa molecular weight marker to higher-order complexes) and an increase in non-native monomeric proinsulin (Figure 8A, red arrows). Additionally, we could resolve proinsulin monomers bearing native disulfide bonds (matching the form of proinsulin secreted into the culture medium (Figure 8A green arrows). When comparing the fraction of proinsulin bearing native disulfide bonds to total proinsulin recovered from the same sample by reducing SDS-PAGE, it was apparent that each of the C-peptide variants exhibited less efficient proinsulin folding, with the Q65R variant appearing least affected, and Q62W and “QV” appearing most affected (Figure 8B). The data support the notion that the folding and trafficking of proinsulin is dependent upon sequence-specific information contained in the amino-terminal portion of the C-peptide structure.

## 3. Discussion

Conserved residues in the C-peptide proximal to the insulin chains do suggest evolutionary pressure to maintain important structural interactions between the two components of the proinsulin molecule [41]. The amino C-peptide sequence A_58_E_59_D_60_L_61_ forms a β-turn that connects via the conserved Q_62_V_63_G_64_Q_65_ to lead into a central flexible Gly/Pro-rich stretch (from G_69_–G_75_) terminated by S_76_, and the C-peptide finally concludes with a well-defined type-III β-turn [36], which is thought to facilitate cleavage at the C-peptide/A-chain junction [50].

Intriguingly, we find that different C-peptide structures altered by distinct recombinant peptide insertions affect proinsulin folding in such a way as to yield a proinsulin secretion efficiency ranging from near-zero (Figure 1C lane 3) to modestly inhibited (Figure 1C lane 2; Figure 1E lane 2) to not impaired at all (Figure 1E lane 3). These phenotypes mirror those of missense mutations in the insulin B- and A-chains, ranging from those that result in severe MIDY [6] to those that produce only a rather mild adult-onset diabetes phenotype [20] and others that are entirely tolerated genetic variants [40]. Interestingly, the many different MIDY mutants exhibit distinct patterns of proinsulin misfolding [38] with distinct degrees of dominant-negative effect on co-expressed WT proinsulin [51] that accompany their distinct levels of clinical severity [20] (such as disease penetrance and age of onset in family members inheriting the variant allele [52]).

MIDY mutations altering the structure of the C-peptide are well known to include frame-shifts, stop codons, alternative splicing, and missense mutations, introducing a cysteine that normally does not exist within the C-peptide, and can dominantly interfere with normal proinsulin disulfide pairing. In contrast, pathogenic non-cysteine C-peptide missense variants are so rare that none have been proven unequivocally [53]. Of the three non-cysteine C-peptide missense variants thus far reported, Edghill and colleagues noted that L68M is probably non-pathogenic, and G84R appears to be a polymorphism of no clinical significance [40].

The report of Schlegel and colleagues of an adult treated for diabetes that might be attributed to heterozygous expression of Q65R [22] inspired us to examine the structural importance of D_60_ [4], plus Q_62_ and V_63_, as well as Q_65_. All of the tested variants exhibited impaired trafficking in cells that do not synthesize endogenous proinsulin (Figure 3B) and still exhibited some impairment (but less so) in β-cells that express abundant endogenous proinsulin (Figure 6B). The trafficking impairment in β-cells is also consistent with diminished insulin generation derived from the variant allele (Figure 6C).

In addition to defective trafficking, the D60Y, Q62W, and “QV” variants each dominantly inhibited the trafficking of co-expressed hPro-CpepMyc (an epitope-tagged proinsulin that, by itself, does not exhibit any defect in protein folding and trafficking; see Figure 1E,F), whereas a dominant-negative impact of the clinically reported Q65R could not be detected (Figure 4). We hypothesize that dominant-negative behavior requires both defective trafficking of the variant and the ability to non-covalently dimerize with co-expressed WT proinsulin. Non-covalent proinsulin dimerization primarily involves association between properly aligned residues in the insulin B-chains of adjacent monomers [54], and by co-immunoprecipitation, this association is unimpaired in proinsulin bearing variant C-peptide (Figure 5). Thus, while we cannot pass judgment on whether the proinsulin Q65R variant can truly trigger dominant, monogenic diabetes in the absence of other risk factors [22], we believe that genetic alterations in the C-peptide structure at or near residues Q_62_ and V_63_ are likely to be a predisposing factor to diabetes in humans.

The mechanism of such impaired trafficking of C-peptide variant proinsulin and co-expressed WT proinsulin appears similar to that detected in MIDY: the C-peptide variants are themselves susceptible to misfolding, including non-native disulfide bonds (Figure 8), which are likely to leave one or more Cys residues unpaired and available to attack bystander WT proinsulin [55,56] to generate a dominant-negative effect. Interestingly, the severity of the dominant-negative effect is related to the variant-to-WT proinsulin expression ratio [48]. Based on our findings, the C-peptide variants that we have studied may be less likely than classic MIDY mutants [38] to bring about a dominant-negative blockade of WT proinsulin trafficking, as the major fraction of hPro-CpepMyc trafficking continued even with a 3:1 variant–WT proinsulin plasmid ratio (Figure 7). Moreover, we observed a partial rescue of the “QV” proinsulin C-peptide variant in the setting in which the WT hPro-CpepMyc protein was in large excess (Figure 7). These data also suggest the possibility that humans, bearing one C-peptide variant allele (and an equally expressed WT allele), might be more sensitive to diabetes than rodents, with one variant allele and three unaffected WT alleles. Indeed, we found that female mice bearing one allele encoding a MIDY mutant R(B22)E did not develop diabetes at all, and males developed diabetes only after high-fat diet feeding [21]. These data do indicate that WT proinsulin-expressing alleles act as suppressors of the diabetes phenotype caused by many misfolded proinsulin variants [57,58].

Altogether, these data suggest that as more human genetic data enters the T2D GWAS portal [4], we anticipate that patients will be identified with enhanced predisposition to diabetes linked to the expression of heterozygous variants located near the amino terminal portion of the C-peptide. Although such patients are less likely to present with the classic forms of MIDY (neonatal diabetes or MODY10), they may be at increased risk of diabetes under conditions of nutrient excess [21] and thus may be incorporated, unknowingly, into the large pool of patients carrying a diagnosis of T2D. 

## 4. Methods

### 4.1. Plasmids and Mutagenesis

Plasmids encoding untagged human proinsulin-WT, proinsulin-D60Y, proinsulin-Q62W, proinsulin-Q65R, and proinsulin-Q62W,V63R (“QV”) in pTarget were generated by QuikChange II site-direct mutagenesis kit (Agilent, Santa Clara, CA, USA). The hPro-CpepMyc construct has been previously described [59], and all other hPro-Cpep tagged constructs (HA, HiBiT, etc.) used the same cloning procedure as that originally described [60]. All constructs were confirmed by direct DNA sequencing.

### 4.2. Antibodies and Human Insulin ELISA

Antibodies in this study include mouse mAb anti-human proinsulin (directed against an epitope in the CA-junction region of PLALEGSLQKRGIV; prepared by Abmart, Berkeley Heights, NJ, USA; kind gift of Dr. M. Liu, Tianjin Medical U. Tianjin China [61]); rabbit anti-Myc (RRID:AB_2921297, Immunology Consultants Laboratories, Portland, OR, USA); and rabbit mAb anti-Hsp90 (RRID:AB_2233307, Cell Signaling, Danvers, MA, USA). For human insulin ELISA, cells lysates were diluted 1:300 in Zero Standard buffer, and the assay was performed with kit 80-INSHU-E01.1 from ALPCO (Salem, NH, USA), with the results calculated with GraphPad Prism 10 software (San Diego, CA, USA).

### 4.3. Cell Lines and Cell Transfections

HEK293T (simply 293T) cells were cultured in DMEM supplemented with 10% fetal bovine serum (FBS) and 1% penicillin/streptomycin. INS1E rat β-cells were cultured in RPMI1640 media supplemented with 10% FBS, 1% sodium pyruvate, 1% HEPES, 1% penicillin/streptomycin, and 0.05 mM 2-mercaptoethanol. All cells were grown in a 37 °C incubator with 5% CO_2_. Cells were seeded in 12-well plates and were transfected at 70–80% confluency using Lipofectamine 2000 (Thermo-Fisher Scientific, Waltham, MA, USA), with fresh media changed at 24 h post transfection. Media were collected at 18 h after refeeding. The cells were washed with ice-cold PBS and lysed in 200 µL RIPA buffer (150 mM NaCl, 1% NP40, 0.5% Na-deoxycholate, 0.1% SDS, 5 mM EDTA, 50 mM Tris pH 7.35) plus protease inhibitor/phosphatase inhibitor cocktail (Thermo-Fisher Scientific, Waltham, MA, USA) on ice for 15 min, scraped, and pipetted up-and-down before transfer to a tube for clarification by centrifugation at 12,000 rpm at 4 °C for 10 min. For co-immunoprecipitation, cells were lysed in IP-lysis buffer (150 mM NaCl, 1% NP-40, 1 mM EDTA, 5% glycerol, 25 mM Tris pH 7.4 plus protease inhibitor/phosphatase inhibitor cocktail).

### 4.4. RNA Isolation, Reverse Transcription and qPCR

RNA was isolated using RNeasy Mini Kit (QIAGEN, Germantown, MD, USA), and concentrations were measured by Nanodrop (Thermo-Fisher Scientific, Waltham, MA, USA). Normalized RNA samples were reverse transcribed into cDNA using SuperScript^TM^ III First-Strand Synthesis SuperMix (Invitrogen, Carlsbad, CA, USA). Quantitative PCR was performed with 200 ng cDNA in 96-well plates with Radiant™ SYBR Green Master Mix qPCR Kit (QS1001 from Alkali Scientific, Ft. Lauderdale, FL, USA). Reactions were run by either QuantStudio3 (Thermo-Fisher Scientific, Waltham, MA, USA) or StepOnePlus (Applied Biosystems, Foster City, CA, USA) with software supplied by the manufacturer. For 293T cells, human ACTB, GAPDH, and 18S were used for reference levels; for INS1E cells, rat HPRT1 was the reference mRNA. Primers used for the genes analyzed in this study are as follows: human proinsulin forward primer: 5′ CAGCCTTTGTGAACCAACAC 3′, reverse primer: 5′ GGTCTTGGGTGTGTAGAAGAAG 3′; human ACTB forward primer: 5′ CATGTACGTTGCTATCCAGGC 3′, reverse primer: 5′ CTCCTTAATGTCACGCACGAT 3′; human GAPDH forward primer: 5′ CCCTTCATTGACCTCAACTACA 3′, reverse primer: 5′ ATGACAAGCTTCCCGTTCTC 3′; human 18s forward primer: 5′ ACCCGTTGAACCCCATTCGTGA 3′, reverse primer: 5′ GCCTCACTAAACCATCCAATCGG 3′; rat HPRT1 forward primer: 5′ CTCATGGACTGATTATGGACAGGA 3′, reverse primer: 5′ GCAGGTCAGCAAAGAACTTATAGCC 3′. Quantitative data were analyzed and plotted by GraphPad Prism 10 (San Diego, CA, USA).

### 4.5. Western Blotting

Total protein in cell lysates was measured by bicinchoninic acid assay. For reducing SDS-PAGE, an identical percentage of cell lysate and media in gel sample buffer containing 200 mM DTT were heated to 95 °C for 5 min and electrophoretically resolved on 4–12% gradient NuPAGE gels. Thereafter, the gels were electrotransferred to nitrocellulose. For non-reducing SDS-PAGE, except where otherwise indicated (e.g., Figure 1D), media and cell lysates were combined (to yield total), heated in gel sample buffer, electrophoretically resolved by straight 12% NuPAGE, and then incubated with 100 mM DTT at 60 °C for 10 min before electrotransfer to nitrocellulose. Primary antibodies were diluted 1:1000 (in Tris-buffered saline with 0.1% Tween-20 plus 5% BSA) and incubated at 4 °C overnight. Horseradish peroxidase-conjugated secondary antibodies were diluted 1:5000 and incubated at room temperature for 1 h before enhanced chemiluminescence with Clarity Western ECL substrate (Bio-Rad, Hercules, CA, USA).

### 4.6. Immunoprecipitation from Cell Lysates

Cells were lysed in IP lysis buffer on ice for 15 min and clarified by centrifugation at 12,000 rpm, 4 °C for 10 min. Cell lysates were immunoprecipitated with anti-Myc-conjugated beads and incubated at 4 °C overnight. After extensive washing, the immunoprecipitates were incubated in gel sample buffer containing 200 mM DTT at 95 °C for 5 min and electrophoretically resolved on 4–12% gradient NuPAGE gels before electrotransfer and immunoblotting with anti-human proinsulin. For co-immunoprecipitation, cells were lysed in IP lysis buffer, as above, and immunoprecipitated with anti-myc antibody-conjugated beads overnight at 4 °C. IP efficiency was calculated by dividing hPro-CpepMyc in the IP samples by hPro-CpepMyc in the initial cell lysate. Co-precipitated proinsulin was calculated by untagged proinsulin in the IP samples divided by untagged proinsulin in the initial cell lysate, normalized for myc-IP efficiency.

### 4.7. Immunofluorescence

INS1E cells were seeded in 8-well chamber-slides. Cells at 70–80% confluency were transfected using Lipofectamine 2000 (Thermo-Fisher Scientific, Waltham, MA, USA). Media were removed at 24 h post-transfection. Cells were fixed with 4% formalin at room temperature for 20 min, permeabilized in TBS plus 3% BSA and 0.4% Triton-X-100 at room temperature for 20 min, and blocked in TBS plus 3% BSA and 0.2% Triton-X-100. The primary antibody was anti-human proinsulin diluted 1:300 in TBS plus 3% BSA and 0.2% Tween-20, incubated overnight at 4 °C. Secondary antibodies were diluted 1:500 in the same buffer, incubated at room temperature for 1 h. Images (60× objective) were captured with a Nikon A1 confocal microscope.

### 4.8. Statistics

Statistical analysis was assessed by unpaired *t*-test, and one-way and two-way ANOVA were employed to compare groups using GraphPad Prism 10 (San Diego, CA, USA). Data are presented as mean ± SD. A *p*-value < 0.05 was considered statistically significant.

## Data Availability

All data and methods for this study are included directly in the manuscript, Figures, Supplementary Figures, and a repository of all experimental metadata uploaded and available at the journal. Materials are freely available upon request.

## Supplementary Materials

The following supporting information can be downloaded at https://www.mdpi.com/article/10.3390/ijms27010483/s1.

## Abbreviations

SNPs: single-nucleotide polymorphisms.
