# Supplementary material for: Structural Communication Between C-Peptide and Insulin Within the Proinsulin Molecule
Source: Int J Mol Sci. 2026 Jan 2;27(1):483. doi: 10.3390/ijms27010483 (PMC12786736; doi:10.3390/ijms27010483)
Supplement: Supplementary file 1 [file ijms-27-00483-s001.zip › ijms-4083082-supplementary.pdf]

Supplemental Figure Legends

Figure S1. Immunoblotting of total proinsulin after nonreducing SDS-PAGE.

Replicate samples like those shown in Fig. 1F (see Methods) were used to quantify the ratio of non-native disulfide-linked proinsulin dimers to proinsulin monomers bearing native disulfide bonds (see green arrows in Fig. 1F); hPro-CpepHA exhibited an increased ratio (an indication of misfolding; n=3 independent experiments; mean ± SD; ****p<0.0001).

Figure S2. Quantification of total recovery of human WT and C-peptide variant proinsulin proteins relative to their respective mRNA levels.

Proinsulin recovery was measured by immunoblotting after reducing SDS-PAGE; mRNA was quantified by qPCR in **A**) 293T cells and **B**) INS1E cells.

Figure S3. Single-channel immunofluorescence images showing human WT and C-peptide variant 'QV' proinsulin in INS1E cells.

Although the anti-human-proinsulin antibody weakly cross-reacts with endogenous rat proinsulin, cells untransfected with human proinsulin constructs fall below image detection.

Figure S4. Quantification of secretion efficiency of WT hPro-CpepMyc co-expressed at different plasmid ratios with untagged WT human proinsulin.

Across a range of plasmid ratios (n=4 independent experiments, mean ± SD), secretion efficiency of hPro-CpepMyc shows no significant change (ns, dark bars) and secretion efficiency of untagged WT human proinsulin also shows no significant change (ns, open bars).

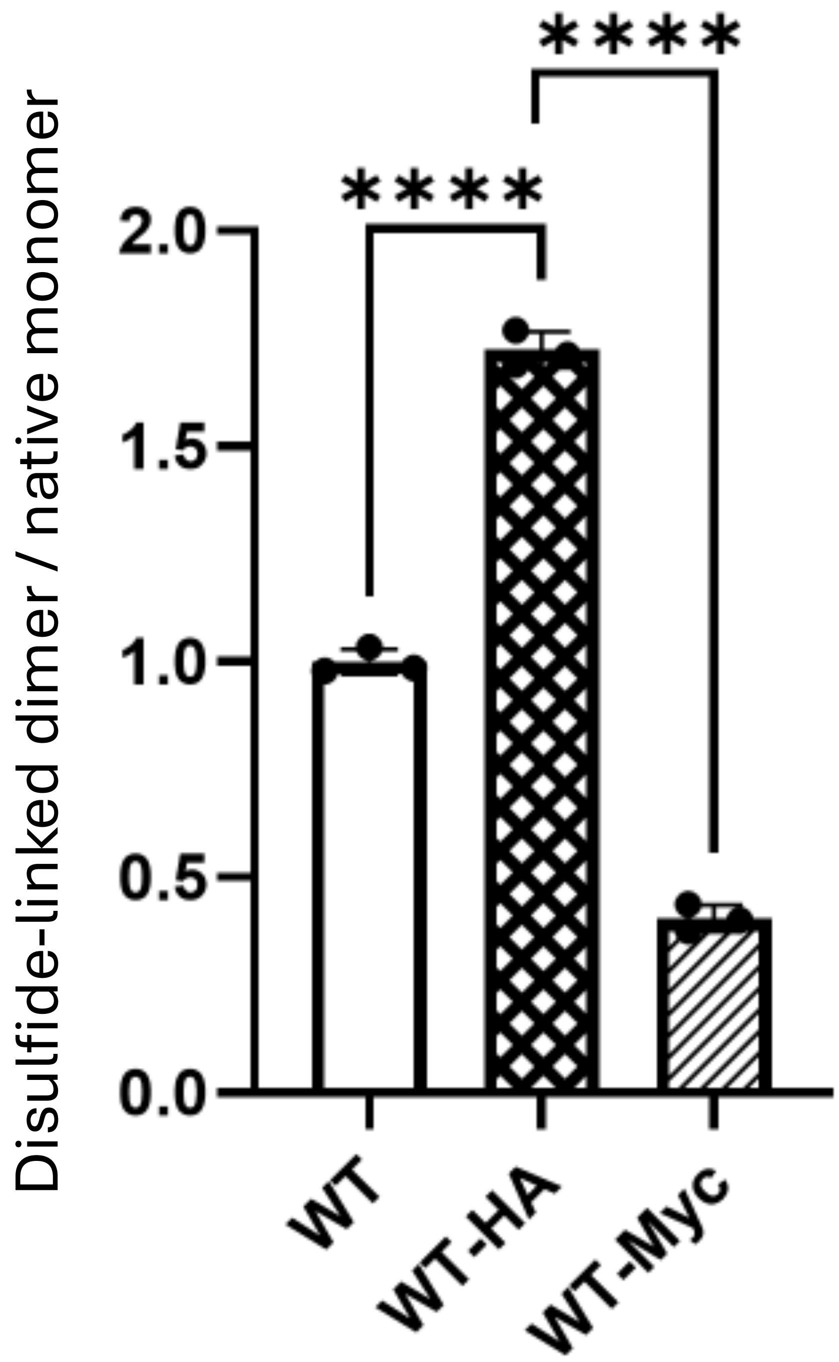

Shao et al., Supplemental Figure S1

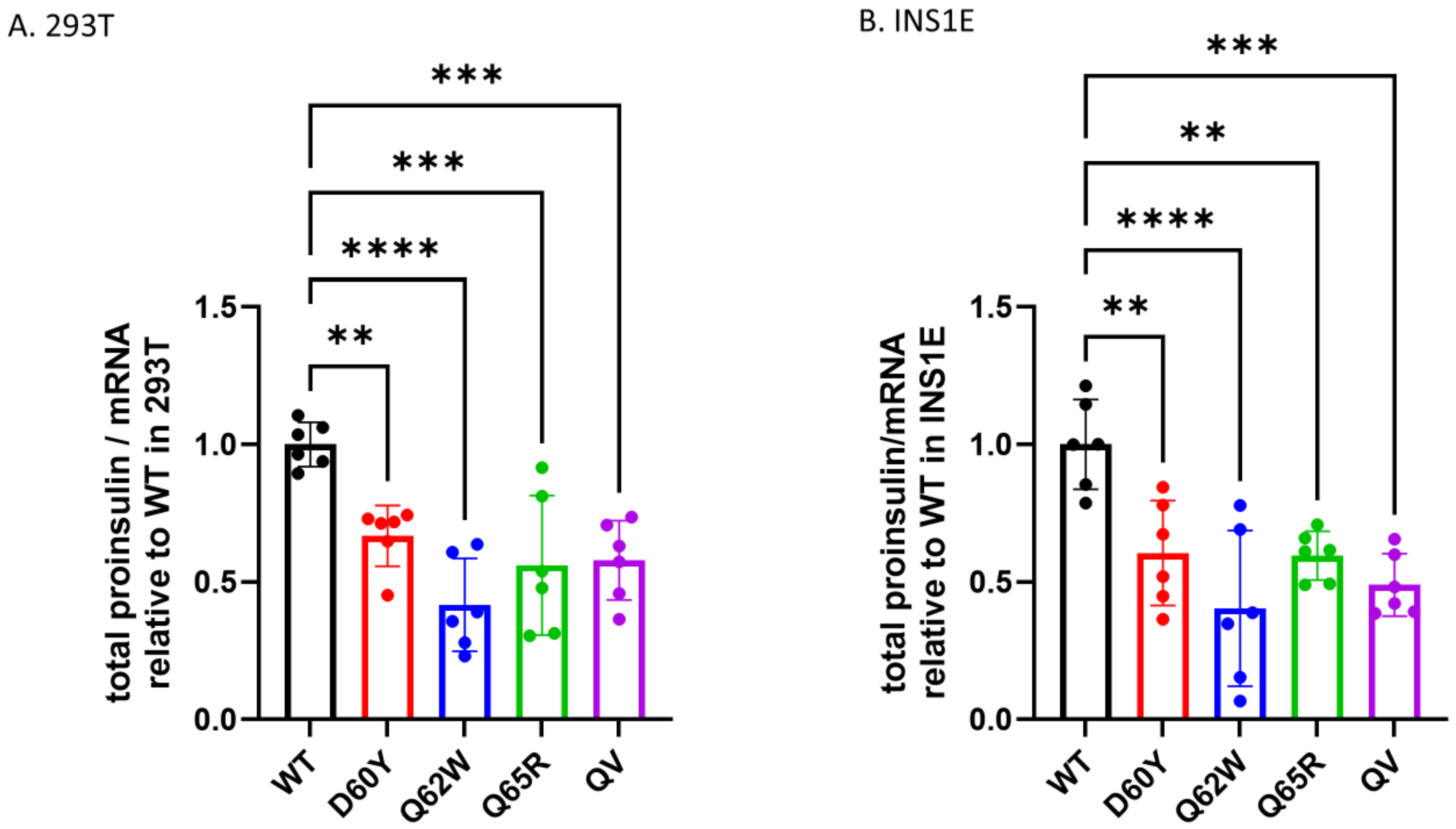

Shao et al., Supplemental Figure S2

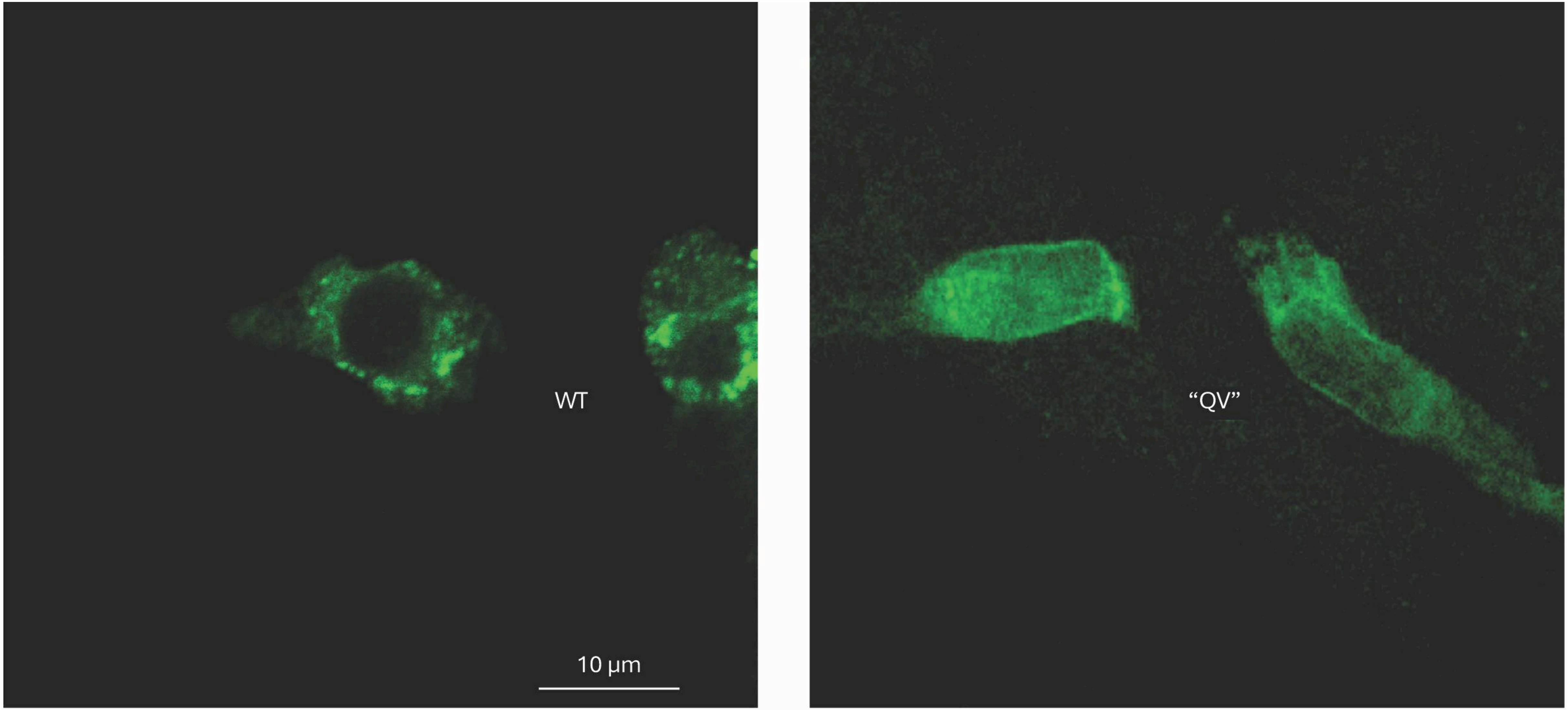

Shao et al., Supplemental Figure S3

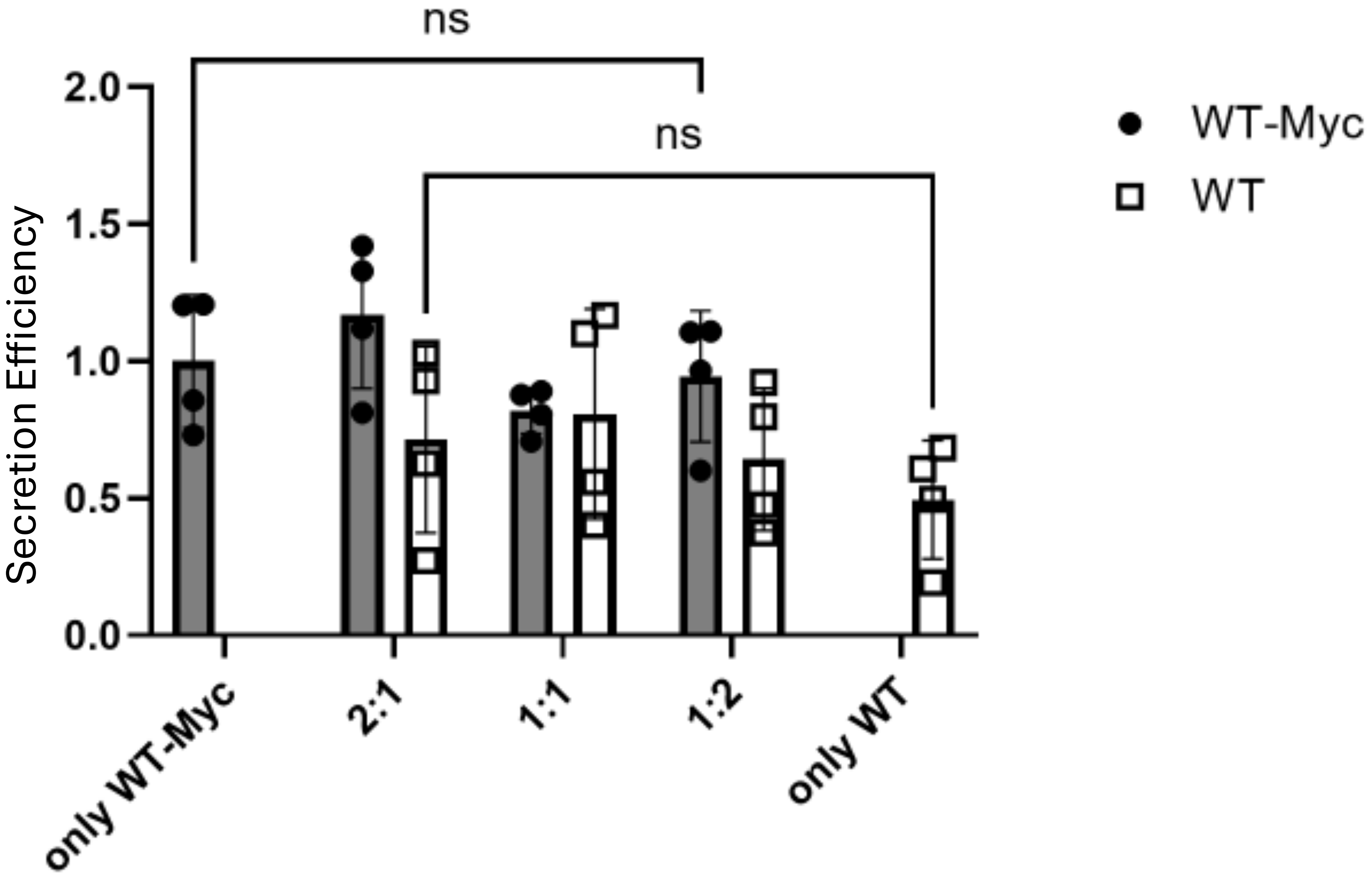

Shao et al., Supplemental Figure S4
